# Extraction, Purification, Structure, and Bioactivities of Polysaccharides from *Glehnia littoralis*: A Review

**DOI:** 10.3390/molecules30214173

**Published:** 2025-10-24

**Authors:** Zhenni Qu, Qi Gao, Menghan Liu, Qiang Zhang, Dianhua Shi, Peng Gao, Yanpeng Dai

**Affiliations:** 1 Shandong Academy of Chinese Medicine, Jinan 250014, China; 13820830896@163.com (Z.Q.); gq20211122@163.com (Q.G.); liu_mh2856@163.com (M.L.); zq809128349@163.com (Q.Z.); shidianhua81@163.com (D.S.); 2 Key Laboratory of Processing Technology and Principle of Honey and Charcoal, State Administration of Traditional Chinese Medicine, Jinan 250014, China; 3 Institute of Pharmacy, Shandong University of Traditional Chinese Medicine, Jinan 250355, China

**Keywords:** glehnia littoralis, extraction, purification, structural characteristics, pharmacological activities, polysaccharides

## Abstract

Ethnopharmacological relevance: *Glehnia littoralis* (GL) is a well-known traditional Chinese medicine used to clear the lungs and benefit the stomach. *Glehnia littoralis* polysaccharides (GLPs) constitute one of the primary active ingredients of GL, demonstrating notable biological activities including immunomodulatory, antioxidant activity, and antitumor effects. Aim of the study: This review aims to provide the latest and the most comprehensive information on GLPs, specifically investigating their extraction technologies, isolation and purification methods, structural characteristics, and pharmacological activities of GLPs. It seeks to lay a foundation for further investigating pharmacological activities and application scope and guide the safe clinical practice of GLPs. Materials and methods: PubMed, Google Scholar, Web of Science, Elsevier, China National Knowledge Infrastructure (CNKI), and other online databases were used to collect literature about extraction, isolation, and purification methods, structural characteristics, and pharmacological activities of GLPs published before January 2025. Results: Polysaccharides are the main active ingredient of GL. Currently, 19 types of GLPs have been extracted. Methods of extracting GL include hot water extraction, ultrasound-assisted extraction, and enzyme extraction. The most frequently used method of separation and purification within GLP is column chromatography, often entailing cellulose column chromatography and ion exchange chromatography. GLPs have various pharmacological activities, including immunomodulatory, antioxidant, and antitumor. Conclusions: While GLPs show promising immunomodulatory and antitumor effects, elucidating their structure–activity relationships is essential for advancing our understanding and requires future research.

## 1. Introduction

*Glehnia littoralis* (GL) is a member of the umbelliferae family, and the desiccated root of GL has long been used as a traditional medicine in China due to its immunoregulatory, antitumor, and antioxidant properties [1,2]. GL was first documented in “Shennong’s Classic of Materia Medica,” which describes its use in fried and honey-processed forms. Meanwhile, GL has also been used in some TCM formulations, such as Yiguanjian Decoction [3] and Yiwei Decoction. GL contains polysaccharides, coumarins, lignans, polyynes, monoterpene glycosides, phenolic acids, volatile oils, and various trace elements [4,5,6], with polysaccharides constituting more than 70% of its composition [7].

Polysaccharides are defined as macromolecular compounds that are composed of numerous monosaccharide molecules connected by glycosidic bonds. These compounds are found in abundance in natural environments. Numerous plant-derived polysaccharides exhibit significant biological activities, such as immunomodulatory, antitumor effects, antiviral properties, radioprotection, hypoglycemic effects, antiaging benefits, and targeted therapy as nano-capsules [8,9,10,11,12,13]. These qualities make polysaccharides valuable in several applications, such as food, medicine, and healthcare [14].

Polysaccharides extracted from GL are molecules with multiple biological activities, including immunomodulation, free radical scavenging, anti-inflammatory, antitumor, and hypoglycemic effects [15,16]. We summarized the extraction and separation techniques, purification procedures, chemical composition, biological activities, pharmacological effects, and the application of GLPs to establish a basis for further investigation of them.

## 2. The Traditional Use of GL

GL has a long history of application, which was first documented in “Shennong’s Classic of the Materia Medica”. Following the publication of “Gleanings from the Compendium of the Materia Medica” and “The Materia Medica Fung Yuen”, it was classified as adenophorae radix and GL. The 2020 edition of the Chinese Pharmacopoeia specifies that the medicinal part of GL is the root, typically harvested in the summer and autumn. The non-medicinal part of GL is further extracted, purified, and dried or boiled to remove the outer skin, after which it is dried and used. The properties of GL are sweet, bitter, slightly cold, and it is effective in nourishing yin, cleansing the lungs, aiding the stomach, and promoting fluid production. It is mainly utilized for lung heat, dry coughs, and coughing with phlegm and blood during labor. GL is primarily cultivated in coastal areas but can also be artificially propagated, which holds significant medical and economic importance. One hundred twenty-six clinical prescriptions of Chinese patent medications contain GL, including Saorilao Lung Clearing and Cough Relieving Capsules, Beishashen Si Wei soup, and Ba Wei Tanxiang powder. Among these, Saorilao Lung Clearing and Cough Relieving Capsules are effective in alleviating cough and promoting expectoration, generating yearly sales in the hundreds of millions of Yuan, and they are utilized in significant amounts. As a medicinal and culinary plant, GL is frequently used in hot and humid regions as a valuable ingredient in soups. At the same time, its stems and leaves serve as esteemed seasonal vegetables that may be brewed into tea. The therapeutic prescriptions of GL comprise Beishashen Baihe soup and Beishashen Xuanshen soup, utilized for treating xerostomia, pharyngitis, and anorexia.

## 3. Preparation Techniques of GLPs

### 3.1. Extraction of GLPs

The biological activity of polysaccharides depends on the extraction method [17], with polysaccharides derived from various extraction techniques demonstrating significant variations in extraction efficiency, monosaccharide composition, molecular weight, glycosidic linkages, and morphological characteristics [18]. These variations influence the biological activity of the polysaccharides, underscoring the necessity of selecting a suitable extraction process [19]. The main methods of extracting polysaccharides can be classified into several categories: hot water extraction [20], acid-assisted extraction [21], alkali-assisted extraction [22], ultrasonic-assisted extraction [23], enzyme-assisted extraction [24], microwave-assisted extraction [25], and multi-method combined extraction [26].

#### 3.1.1. Solvent Extraction

The solvent extraction technique requires the selection of suitable solvents predicated on the solubility, molecular weight, and ionic properties of polysaccharides. Commonly used solvents comprise water and acid–base solutions. The polysaccharide components with varying molecular weights are precipitated using different concentrations of ethanol. The water extraction method is favored for its safety and environmental friendliness, making it the most prevalent technique for extracting GLPs. Shen [27] obtained crude GLPs via water extraction. The principal parameters affecting the process were the frequency of extraction, extraction temperature, duration of extraction, and the material-to-water ratio. The duration of extraction was found to significantly influence the polysaccharide extraction rate more than the extraction temperature and the extraction frequency. The extraction rate of crude GLPs was determined to be 15.59% (on a dry weight basis of the raw material) under the conditions of three extraction cycles, a temperature of 90 °C, a duration of four hours, and an optimal material-to-water ratio of 1:30. The validation experiment results were higher than those from the orthogonal test. However, the increased temperature and extended duration of this method may jeopardize the stability of GLPs [28]. GLPs were extracted utilizing a water extraction technique, with the ethanol concentration generally sustained between 80 and 95%. The polysaccharide extraction rate ranged from 10.78% to 60.15%. The wide variation in extraction rates is due to the process conditions affecting the composition and molecular weight of the polysaccharides. The variations in extraction outcomes are related to differences in process parameters, raw materials, and analytical techniques used for determination.

#### 3.1.2. Acid-Assisted and Alkali-Assisted Extraction

Acid-assisted extraction has been established as an effective method for producing low molecular weight polysaccharides with enhanced biological activity [29]. Comparative studies on GLPs extraction efficiency have demonstrated that acid-assisted extraction (0.3 mol/L HCl, 1:30 solid–liquid ratio) at 50 °C with dual extraction cycles produces maximal polysaccharide yields [22]. The alkali-assisted extraction method primarily utilizes 0.1~1 mol/L NaOH to extract acidic polysaccharides, facilitate component dissolution, and increase yield. However, the alkali-assisted extraction technique may induce glycosidic bond degradation that compromises bioactivity. Furthermore, the alkali-assisted extraction solution exhibits significant viscosity, complicating the filtration process.

#### 3.1.3. Ultrasonic-Assisted Extraction

Ultrasonic-assisted extraction is a technique that leverages the mechanical, thermal, and cavitation effects of ultrasound in a liquid medium to disrupt cells and solubilize polysaccharides [30,31,32]. This procedure offers several benefits, including swift extraction velocity, elevated efficiency, and a short extraction duration. The final product, GLP80-1, was obtained by a series of ultrasound-assisted extraction procedures. The extraction temperature was set at 74 °C, the extraction duration was 27 min, the liquid-to-feed ratio was 103 mL/g, the ultrasonic power was 198 W, and ethanol precipitation was employed. The yield was 4.34% [33,34].

#### 3.1.4. Microwave-Assisted Extraction

Microwave-assisted extraction employs high-frequency electromagnetic waves to induce cell rupture, facilitating the fast release of intracellular active compounds in conjunction with organic solvents [35]. While this method demonstrates particular efficacy in GLP isolation, operational optimization studies have identified critical parameters: immersion for 30 min, microwave power set at 800 W, microwave irradiation duration of 100 s, a solid–liquid ratio of 1:30 (g/mL), a crushed particle size of 100 mesh, and extraction conducted three times, achieving 39.3% crude polysaccharide yield. The content of polysaccharides in the extract is 65.4%. Compared with the test sample with the highest polysaccharide yield, the polysaccharide yield of the verification test was slightly lower, but the difference was not significant [36]. It should be noted that this approach has several drawbacks, including uneven heat transfer, potential damage to polysaccharide structures due to high temperatures, and prohibitive costs for large-scale production [37].

#### 3.1.5. Enzymatic Extraction

Enzymatic extraction is an effective and eco-friendly technique that employs cellulases, pectinases, and proteases to disrupt cellular structures, thereby facilitating the release of polysaccharides [13]. This method necessitates stringent extraction conditions, while suboptimal conditions can alter enzyme protein conformation and properties, consequently affecting the composition ratio of polysaccharides. Therefore, the enzymatic extraction of polysaccharides must be meticulously regulated by enzyme conditions [38,39]. Cellulase is the predominant technique for extracting GLPs. Certain researchers employed response surface analysis to refine the extraction process of GLP-E, establishing optimal conditions: an enzyme temperature of 70 °C, an enzyme duration of 3 h, a liquid-to-solid ratio of 30:1 (mL/g), an enzyme additive concentration of 3.0%, and a cellulase to papain mass ratio of 3:1, Carried out three verification experiments, resulting in a GLP extraction rate of (22.04 ± 0.23)%. The difference from the predicted value of 22.30% is 0.25%, indicating that using this model for the process optimization of the extraction of GLPs has certain practical operability. The difference from the predicted value of 22.30% is 0.25%, indicating that using this model for the process optimization of the extraction of GLPs has certain practical operability. The difference from the predicted value of 22.30% is 0.25%, indicating that using this model for the process optimization of the extraction of GLPs has certain practical operability [40].

#### 3.1.6. Multi-Method Joint Extraction

Multi-method joint extraction optimally utilizes the strengths of many extraction techniques, compensates for their individual limitations, reduces extraction time, and enhances polysaccharide yield. Researchers choose cellulase and ultrasonic-assisted extraction techniques to obtain GLPs. The findings from the single-factor analysis were consolidated, and the Box–Behnken response surface methodology was employed to ascertain the optimal extraction parameters for polysaccharides: enzyme digestion duration of 112 min, material-to-liquid ratio of 1:30 g/mL, ultrasound power of 210 W, ultrasonic duration of 41 min, ultrasonic temperature of 65 °C, and enzyme concentration of 2%. Under these conditions, the yield of GLP was 39.58 ± 0.90%. Three verification tests were conducted simultaneously. The result showed an error of 1.62% compared to the predicted value of 41.25%, which was less than 2%, indicating that this model can effectively predict the extraction process of GLPs [41]. Table 1 delineates the various extraction methods and outcomes for GLPs.

### 3.2. Isolation and Purification of GLPs

The isolation and purification of polysaccharides are essential for investigating their structure and biological activity. At the same time, higher polysaccharide purity is related to the enhanced stability of its structure and activity. The polysaccharides obtained through hot water extraction and ultrasonic-assisted extraction are often crude, frequently containing contaminants such as proteins, colors, and tiny molecular components, which can influence the structural analysis and biological activity of GLPs. Consequently, they must first undergo purification through deproteinization, decolorization, and impurity removal, followed by grading to obtain homogeneous polysaccharides. Small molecules in crude polysaccharides are typically eliminated through multiple alcohol precipitations, while proteins are primarily removed using the Sevag or enzymatic methods. Decolorization is generally achieved via activated carbon adsorption, H_2_O_2_ oxidation, and macroporous resins’ adsorption and decolorization capabilities. Sequential precipitation processes, metal complexation techniques, molecular sieve chromatography, ion exchange chromatography, and column chromatography are classification and purification methods to isolate a single component with enhanced purity [51].

Enzymatic methods, Sevag process, trifluorotrichloroethane, trichloroacetic acid, and tandem anion and cation techniques are frequently employed to deproteinize GLPs [52]. The Sevag reagent (trichloromethane-water-saturated n-butanol in a 4:1 ratio) is extensively utilized to eliminate proteins from crude polysaccharide extracts. This process relies on the principle that proteins are denatured in organic solvents like trichloromethane, resulting in a gel formation that can be separated through centrifugation [53]. The efficacy of protein elimination with the Sevag method is comparatively inadequate, necessitating multiple repetitions to achieve complete protein removal. This approach is mild and successfully prevents the degradation of polysaccharides. Upon deproteinization of the crude polysaccharide, it can be further purified and fractionated by gel column chromatography and other techniques, yielding polysaccharides with a consistent relative molecular mass. Research indicates that the savage procedure, used thrice, can be utilized until no discernible denatured protein remains at the interface of the trichloromethane-water-saturated n-butanol layers. The color of polysaccharides is attributed to phenolic compounds in crude polysaccharides, which will influence their purity. Thus, it is necessary to decolorize the solution containing polysaccharides. The commonly employed decolourization materials encompass activated carbon, macroporous resin, ion exchange resin, and hydrogen peroxide. Activated carbon adsorption is the principal procedure for the decolourization of GLPs. The separation and purification techniques are essential for polysaccharides. Acquiring polysaccharides with consistent molecular weight and polarity is vital for their structural analysis. The prevalent methods for separating and purifying polysaccharides include gradient precipitation, column chromatography, and membrane separation [54], in which column chromatography is the predominant purification technology for GLPs. However, it is not commonly used to separate GLPs, and there is no relevant report on the separation of GLP by the membrane separation method. The type of column chromatography includes cellulose column chromatography, ion exchange chromatography, gel filtration chromatography, affinity chromatography, high-pressure liquid chromatography, and other chromatographic techniques. Ion exchange chromatography and gel filtration chromatography are two predominant techniques widely employed in practice. Among ion exchange column packings, DEAE-cellulose represents one of the most extensively utilized materials, particularly effective for the separation of neutral and acidic polysaccharides. Similarly, DEAE-Sepharose is also widely applied, with Sephadex being the most commonly used gel agent. The molecular weight-dependent separation of polysaccharides exhibits a direct correlation with matrix crosslinking density, where highly cross-linked stationary phases demonstrate superior resolution for lower molecular weight saccharide fractions. The compositional analysis of GLPs employs multiple chromatographic techniques, with gas chromatography, high-performance gel permeation chromatography, and high-performance liquid chromatography representing the most widely adopted analytical methods in current research practice. The researchers employed ion exchange chromatography and gel filtration column chromatography to purify the crude polysaccharide, ultimately obtaining homogeneous GLPs through the DEAE-cellulose-52 column [55]. The researchers employed diethyl aminoethyl (DEAE)-cellulose-52 column to isolate neutral and acidic polysaccharides, followed by molecular weight-based fractionation using Sephadex G-100 gel filtration [19]. This purification protocol yielded the target polysaccharide GLP80-1 with a yield of 4.34% [34]. The separation and purification process of polysaccharides significantly influences the yield.

Current purification processes are time-consuming and laborious, and they may lead to loss of polysaccharide activity. Systematic optimization of these protocols towards high-efficiency, low-energy methodologies is a priority for future research. Future research should move towards goal-oriented purification strategies, such as supercritical fluid extraction (SFE) [56], deep eutectic solvents (DES) [57] and co-extraction methods, or preparative high-performance liquid chromatography (Prep-HPLC). The use of Prep-HPLC in conjunction with on-line activity assays (e.g., DPPH or cytokine secretion assays) allows for direct targeting and isolation of the key active components, bypassing lengthy traditional processes.

## 4. Structural Characteristics of GLPs

The structural diversity of polysaccharides fundamentally governs their biological activity profiles, necessitating precise structural elucidation [58]. The structural characterization of polysaccharides includes the content of monosaccharides, relative molecular mass, types of glycosidic linkages, and structural conformation [59]. IC, FTIR, HPLC, HPGPC, GC, NMR, and other techniques are routinely employed to analyse and identify the structural characteristics of polysaccharides.

### 4.1. Purity of GLPs

The analysis of polysaccharide purity is crucial to examining their structural properties. The phenol-sulfuric acid approach is presently employed to quantify GLPs, whereas UV analysis is utilized to detect leftover proteins, nucleic acids, and other contaminants. Researchers have indeed applied the phenol-sulfuric acid technique for GLP determination. Glucose served as the reference for quantifying the level of soluble polysaccharides. The findings indicated that the overall concentration of soluble polysaccharides exceeded 80%, with a content range from 81.28 ± 0.02% to 89.10 ± 0.10%.,which indicated a significant concentration of soluble sugars in the GLPs. The *p* value is 0.002, which is highly significant [48]. Researchers analyzed the purity of GLP90-2 using HPGPC and observed a sharp, narrow peak, signifying that GLP90-2 is a homogenous polysaccharide of high purity [60]. The literature indicates that the purity of polysaccharides was assessed using a UV spectrophotometer at 280 nm and 260 nm, showing no absorption, which suggests the absence of proteins and nucleic acids [13,61,62].

### 4.2. Molecular Weight

The relative molecular mass is a significant physical property of polysaccharides. The biological activity of polysaccharides is intimately connected to their molecular weight. GPC, HPGPC, and HPSEC can ascertain the relative molecular mass of polysaccharides.

The molecular weight of GRP-1 is 23.01 kDa, while GRP has a molecular weight of 13.3 kDa [63,64]. For GLP90-2, high-performance gel permeation chromatography (HPGPC) coupled with multi-angle laser scattering and refractive index detection yielded a molecular weight of 7.76 × 10^3^ g/mol and a polydispersity index of 1.06 [60]. The molecular masses of GLPs showed considerable variation (10.24~4626.41 kDa), likely due to methodological differences in their extraction, separation, purification, and analytical procedures.

### 4.3. Composition and Chemical Structure of Monosaccharides

**Figure 1:**
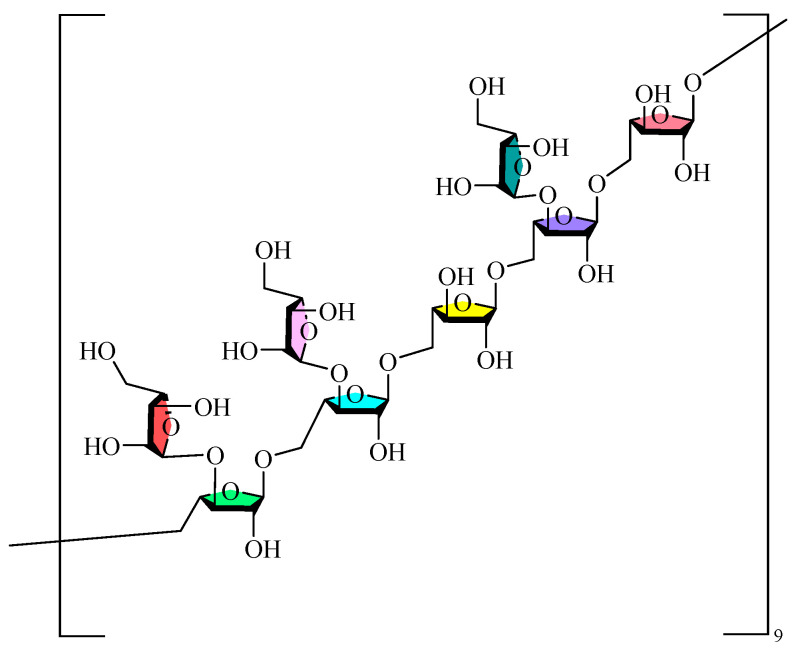
Possible structural repeat units of GLPs.

Monosaccharides serve as the fundamental units of polysaccharide macromolecules, and the investigation of their composition is a crucial step in examining their physicochemical properties and structural characteristics. The prevalent techniques for qualitatively or quantitatively examining polysaccharides are HPLC, GC, CE, and thin-layer chromatography (TLC). The monosaccharides contained in GLPs are mainly Glc, Man, GlcA, Gal, Ara, and Rha, in addition to small amounts of Xyl and Fuc. The composition and concentration of the monosaccharides in GLPs are influenced by several factors, including cultivar, geographic origin, year of cultivation, harvest timing, and extraction and purifying techniques. These findings confirm that GLPs’ monosaccharide profiles are significantly affected by these key parameters. The current research on the sugar chain structure of GLPs mainly focuses on the types and arrangements of monosaccharides, along with their interconnections. Monosaccharides serve as the building blocks of polysaccharides, interconnected by characteristic glycosidic bonds (α-1,4-, β-1,4-, and α-1,6- linkages). Glycosidic bonds are typically characterized through a series of chemical and physical analyses, including methylation reactions, Smith degradation, acid hydrolysis, and periodate oxidation. These methods can ascertain the monosaccharides composition, glycosidic bond types/ratios, positional characteristics, and branching patterns in GLPs. Infrared (IR), Nuclear Magnetic Resonance (NMR) spectroscopy, Gas Chromatography (GC), Mass Spectrometry (MS), H-HCOSY, and other physical methods provide detailed information about glycosidic bond conformations, substituent groups, and attachment sequences [65,66]. Moreover, biological approaches, like glycosidase hydrolysis, are essential for examining its structure when required. Researchers have employed HILIC LC-MS to ascertain the fracture pattern of the polysaccharides in GLPs, which indicated that GLP hydrolyzed linear gluco-oligosaccharides with 1,4-glycosidic linkages [63]. The integration of methylation reactions, periodate oxidation, and Smith degradation demonstrated that the GRPs comprised 1→3, 1→6, 1→4,6, and 1→linked glucans in molar ratios of 1.1:12.8:1:1.3, with defined repeating units [67]. Structural characterization of GLP90-2 identified it as a highly branched arabinoglycan, characterized by a leading chain of →5)-α-L-Ara*f*-(1→ and a branched →3,5)-α-L-Ara*f*-(1→ at the O-3 position, featuring an α-L-Ara*f*-(1→ terminal residue [60]. Furthermore, it was determined that both GL-100 and GL-103 are linear glucans connected by α-(1→4) glycosidic linkages [68]. Figure 1 illustrates the potential structural repetition units of the inferred GLPs.

GLPs possess unique structural characteristics and biological advantages that distinguish them from other well-studied medicinal polysaccharides. The purified fractions of GLPs are mainly composed of glucose, along with small amounts of mannose, glucuronic acid, rhamnose, galactose, and arabinose, and contain α-glycosidic bonds. In comparison with Dendrobium polysaccharides [69]-which are rich in mannose and glucose and contain β-(1→4) glycosidic bonds—the α-glycosidic bond configuration and glucose-dominant composition of GLPs constitute their unique structural signature. Although nearly 20 GLPs have been reported, the conformational relationships of their effects need to be further elucidated, and the research in this area is still in the preliminary stage. Table 2 displays the purity, relative molecular mass, type of glycosidic bond, and monosaccharide makeup of GLPs. Table 3 presents the structural information and detection methodologies of GLPs.

## 5. Pharmacological Activities of GLPs

### 5.1. Immunomodulatory Effects

Natural polysaccharides exert immunomodulatory effects via multiple mechanisms and targets. Polysaccharides can modulate cytokine production and release, affect immune cell contacts and signaling pathways, and control immune cell differentiation and functionality, thereby maintaining the stability and equilibrium of the immune system [74]. In vitro immunoreactivity experiments demonstrated that GLP significantly enhanced the proliferation of RAW 264.7 macrophages at concentrations ranging from 0.5 to 250 μg/mL (*p* < 0.05). The experimental results demonstrated that GLP exerted maximal proliferative effects on RAW 264.7 macrophages at concentrations of 10 and 50 μg/mL. Furthermore, at lower concentrations (0.5, 1, 10 μg/mL), GLP effectively mitigated the lipopolysaccharide (LPS) induced hyperactivation of RAW 264.7 macrophages, indicating its potential as an immunomodulator [41]. Experimental studies have demonstrated that GLPs significantly enhanced the NK cell cytotoxicity of spleen and promoted T lymphocyte activation in hyperthyroid yin-deficient mice, concurrently elevating serum immunoglobulin (IgM and IgG) levels. Simultaneously, the polysaccharide fractions, including GRP-1~3, GLP-D1, D2, GLP, GLP80-1, and GRP, exhibited proliferation-enhancing effects on murine splenic lymphocytes. Furthermore, GLP and GLP80-1 exhibited dose-dependent stimulation of nitric oxide production (0.5–250 mg/mL) and significantly upregulated TNF-α and IL-6 secretion in macrophages. These findings collectively suggest that the polysaccharide portions of GRP effectively stimulated lymphocyte proliferation, whereas GLP and GLP80-1 considerably enhanced the production of TNF-α and IL-6 in macrophages [2,34,63,71].

These investigations indicate that GLPs possess potential immunomodulatory properties and may serve as immunomodulators in functional foods, which are characterized by both high efficacy and low toxicity. Mechanistically, GLPs exert immunomodulatory effects through multiple pathways, including macrophage activation and cytokine regulation, as described above [34].

### 5.2. Antioxidant Effects

Oxidative stress is intricately linked to numerous diseases, such as cardiovascular disease, cancer, and neurodegenerative disorders [75,76,77]. Researchers employed the graded precipitation technique to purify GLP, revealing that the GLP-50 exhibited notable antioxidant activity. The GLP-50 possessed the highest concentration of galacturonic acid and an elevated content of protein relative to other graded precipitation polysaccharide fractions, suggesting that galacturonic acid and protein levels in polysaccharides may influence their antioxidant activity. The results showed that the ability to scavenge DPPH radicals was related to the molecular weight, and the higher the molecular weight, the stronger the ability to scavenge DPPH radicals, but they were all lower than the scavenging ability of the positive control VC (IC50 of 0.005 mg/mL) [40]. The scavenging ability of GLP-30, GLP-50, and GLP-70 on DPPH free radicals increased gradually with increasing sugar concentration, showing concentration dependence and the smallest IC50 value, indicating that they had the strongest scavenging ability. The scavenging capacities of these three polysaccharides and vitamin C against DPPH free radicals were as follows: vitamin C (0.005 mg/mL) > GLP-50 (0.123 mg/mL) > GLP-70 (0.364 mg/mL) > GLP-30 (1.822 mg/mL). The GLPs were lower than the IC50 value of the positive control vitamin C. The above results indicated that GLPs have certain free radical scavenging ability [1]. Experimental evidence confirms that GLPs exhibit significant antioxidant activity. Nevertheless, the precise mechanism of action requires additional investigation. Researchers have isolated and purified a GLP and synthesized GLP-NPs using the nanoprecipitation technique. Then, the GLP-NPs served as a reducing agent to produce GLP-NPs-AgNPs. The chelation efficiency of nanosilver with polysaccharides could reach 67.5%. The IC_50_ values for hydroxyl radicals were 2412.8 μg/mL for GLP, 1126.5 μg/mL for GLP-NPs, 590.3 μg/mL for GLP-NPs-AgNPs, and 28.9 μg/mL for Vc. Notably, the GLP-NPsAgNPs demonstrated superior free radical scavenging and antioxidant activities compared to GLP and GLP-NPs [55]. This may be attributed to the effective antioxidant properties of polysaccharides, as their lone pair of electrons interacts with free radical scavengers, thereby neutralizing free radicals. Concurrently, GLP-NPs-AgNPs exhibit enhanced antioxidant activity due to their increased specific surface area, which enables greater adsorption of free radical scavengers. Furthermore, the coordinated interaction between polysaccharides and metal ions, combined with synergistic electrostatic effects, facilitates the generation of efficient radical-scavenging complexes [78].

In summary, GLPs possess considerable antioxidant activity, which can mitigate oxidative stress-induced damage, safeguard cells from free radicals, preserve normal cellular function, and contribute to preventing and treating diseases associated with oxidative stress.

### 5.3. Antitumor Effects

In recent years, the investigation of traditional Chinese medicine for tumor treatment has gained prominence. Chinese medicines typically exert anti-tumour actions by modulating tumour gene expression, suppressing cancer cell growth, and causing apoptosis and autophagy in tumour cells. Polysaccharides can impede the proliferation of cancer cells by modifying macrophage activity, enhancing the synthesis of anticancer antibodies, elevating nitric oxide levels, and disrupting free radicals [79,80]. The monomer vanillic acid 1-O-[β-D-apiofuranosyl-(1→6)-β-D-glucopyranoside] ester, isolated from herbs, exhibited specific inhibitory efficacy on tumour necrosis factor production [33]. In a recent study, researchers utilized the MTT method to assess the inhibitory effect of GLPs on the proliferation of A549 cells. The findings revealed that GLPs exhibited the strongest inhibitory effect on A549 cell proliferation at a mass concentration of 380 μg/mL. Additionally, GLPs exhibit significant inhibitory effects on A549 cell migration while concurrently inducing tumor cell apoptosis, demonstrating potent anticancer efficacy. The underlying mechanism may be associated with the downregulation of proliferating cell nuclear antigen (PCNA), leading to cell cycle arrest in the S and G2/M phases.

**Figure 2:**
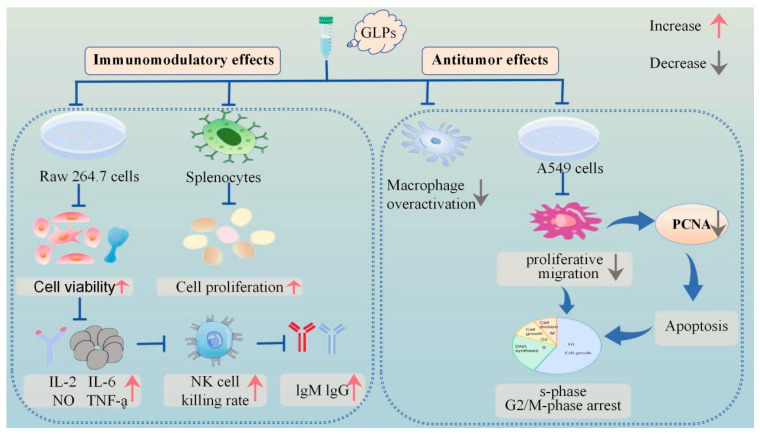
Schematic representation of immunomodulatory and antitumour activities of GLPs.

GLP90-2 suppressed tumorigenesis and metastasis in a zebrafish model, which validated its angiogenesis inhibition effects. Meanwhile, the results showed that GLP90-2 facilitates the maturation of DC2.4 cells and macrophages while augmenting the expression of immune-related cytokines. Concurrently, GLP90-2 demonstrated robust binding affinity to PD-1, potentiating immune system activation. Mechanistic investigations further revealed its anti-angiogenic activity in a transgenic zebrafish model, mediated through modulation of the VEGF/VEGFR-2 signaling pathway. SPR assays quantitatively validated the direct molecular interaction between GLP90-2 and VEGF. These findings collectively suggest that the observed suppression of angiogenesis and tumor progression may mechanistically depend on GLP90-2/VEGF binding events. Notably, in vivo studies confirmed the dual-functional therapeutic efficacy of GLP90-2, exhibiting both potent immunomodulatory properties and anti-neoplastic effects, thereby underscoring its translational potential as a novel cancer immunotherapeutic candidate [42]. Figure 2 graphically illustrates the immunological and anti-tumor effects of GLPs. The comprehensive biological actions of GLPs are presented in Table 4.

In summary, GLPs demonstrate anti-tumour effects via multiple mechanisms, including the inhibition of tumour cell proliferation, induction of tumour cell apoptosis, and suppression of tumour angiogenesis. It must be emphasized that the structure–activity relationships underlying these effects still need to be clarified. Although nearly 20 bioactive GLP structures have been reported, research in this area is still in its preliminary stages.

## 6. Summary and Prospects

This review systematically summarizes the significant progress achieved in the research of GLPs, covering aspects such as extraction, purification, structural characterization, and diverse bioactivities. Traditional methods, particularly water extraction and alcohol precipitation, remain fundamental. Although auxiliary techniques like ultrasound and microwave irradiation have improved efficiency, the mainstream purification—involving decolorization, deproteinization, and chromatographic separation—often constitutes a protracted, multi-step process that risks compromising the native bioactivity of GLPs.

GLPs possess unique structural characteristics and biological advantages that distinguish them from other well-studied medicinal polysaccharides. The purified fractions of GLPs are mainly composed of glucose, along with small amounts of mannose, glucuronic acid, rhamnose, galactose, and arabinose, and contain α-glycosidic bonds. In comparison with Dendrobium polysaccharides [67]-which are rich in mannose and glucose and contain β-(1→4) glycosidic bonds-the α-glycosidic bond configuration and glucose-dominant composition of GLPs constitute their unique structural signature. Although nearly 20 GLPs have been reported, the conformational relationships of their effects need to be further elucidated, and the research in this area is still in the preliminary stage. In terms of anti-inflammatory activity, Lonicera japonica polysaccharides regulate the expression of TNF-α and IL-6 through the NF-κB signaling pathway [74]. In contrast, preliminary research data on GLPs indicate that they may exert anti-inflammatory effects by regulating macrophage polarization, without inducing side effects such as splenic tissue changes caused by high-dose polysaccharides. Furthermore, the immunomodulatory efficacy of GLPs is superior to that of Chrysanthemum morifolium polysaccharides [74]. These studies suggest that GLPs have potential research value and can complement the therapeutic spectrum of traditional medicinal polysaccharides.

To translate fundamental knowledge into impactful applications and mechanistic understanding, future research must adopt more sophisticated and targeted strategies. For instance, integrated approaches combining Response Surface Methodology (RSM) with Artificial Neural Networks (ANN) should replace traditional one-factor-at-a-time optimization. This strategy can model complex parameter interactions (e.g., temperature, time, power) to predict global optimum conditions for simultaneously maximizing yield and target bioactivity. Advanced chromatographic systems such as preparative Ultra-High Performance Liquid Chromatography (prep-UHPLC) could be employed to directly and efficiently isolate polysaccharide fractions.

Multidimensional NMR spectroscopy (e.g., 2D COSY, NOESY, HSQC) should be utilized to resolve glycosidic linkage patterns and sequences. This can be combined with enzymatic digestion and mass spectrometry to obtain fine structural details and verify NMR assignments. Structure-function correlations must be established by systematically linking specific structural motifs (e.g., monosaccharide composition, glycosidic linkage types, degree of branching, and molecular weight) with quantitative bioactivity data to construct predictive Structure–Activity Relationship (SAR) models.

This “bionic degradation” strategy aims to elucidate the formation of active forms of GLPs in vivo. This approach is critical for understanding how large molecular weight GLPs are degraded into active fragments in the body, although significant challenges remain in this area. The in vivo mechanism of action can be revealed through a bionic degradation strategy:

Simulated Digestion: Sequentially treat GLPs with simulated gastric and intestinal fluids to mimic the human digestive process in vitro.

Metabolite Profiling: Analyze the resulting oligosaccharide fragments using UPLC-Q-TOF-MS/MS to identify cleavage sites (e.g., of alpha-glycosidic bonds) and characterize the degradation products.

Bioactivity Screening: Isolate the predominant oligosaccharide fragments via preparative UPLC and subject them to relevant in vitro bioassays (e.g., immunomodulation on macrophages) to pinpoint the core active structures.

**Figure 3:**
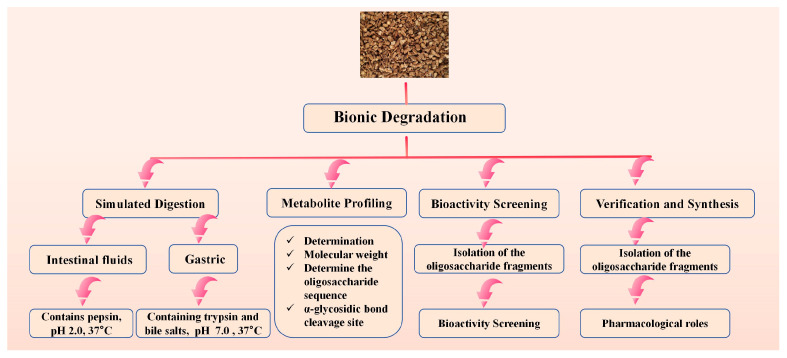
Bionic Explanation Route Map.

Verification and Synthesis: Chemically synthesize the identified active oligosaccharides to confirm their pharmacological roles. Figure 3 shows the bionic explanation route map.

It should be particularly noted that although GL is clinically used in the treatment of gastritis and lung cancer, the clinical application of isolated GLPs for these diseases has not been reported. All current studies on the anti-inflammatory and anti-tumor activities of GLPs are in the preclinical stage. Therefore, exploring the role of GLPs in specific disease models, such as gastritis and lung cancer, is crucial for explaining the traditional efficacy of GL and developing a new generation of polysaccharide-based drugs.

Future developments could focus on creating multifunctional wound dressings based on GLPs. Exploring GLPs as natural stabilizers and active ingredients in cosmeceuticals, leveraging their antioxidant properties, or engineering them into nanoparticulate drug delivery systems for targeted cancer therapy are also promising avenues.

## Figures and Tables

**Table 1:** Methods and results of extraction of GLPs.

Method	Optimization Factors	Optimization Methods	Other Extraction Conditions	Results			Ref.
				Degree Ranking	Optimization Results	Extraction Rate (%)	
Hot water extraction	Extraction times (A) Extraction temperature (B) Extraction time (C) Solid–liquid ratio (D)	Univariate investigation Orthogonal design method	95% ethanol	C > B > A > D	A (3) B (90 °C) C (4 h) D (1:30)	15.59	[2,27,42,43,44,45,46]
	Extraction times (A) Extraction temperature (B) Solid–liquid ratio (C)	Not Reported	Centrifugation (3000 r/min, 15 min)	Not Reported	A (2) B (94.9 °C) C (1:20)	Not Reported	[2]
	Extraction times (A) Extraction temperature (B) Extraction time (C)	Not Reported	Centrifugation (4000 r/min, 10 min)	Not Reported	A (3) B (90 °C) C (3 h)	Not Reported	[42]
	Extraction temperature (A) Extraction time (B) Solid–liquid ratio (C)	central composite design and response surface methodology	Extraction times (2)	C > A > B	A (94.9 °C) B (2 h) C (1:25)	10.78	[43]
Acid-assisted extraction	Extraction time (A) Solid–liquid ratio (B) Extraction temperature (C)	Not Reported	Extraction times (2); HCl (0.3 mol/L)	Not Reported	A (2 h) B (1:30) C (50 °C)	39.62	[22,47]
Alkali-assisted extraction	Extraction time (A) Solid–liquid ratio (B) Extraction temperature (C)	Not Reported	Extraction times (2); NaoH (0.3 mol/L)	Not Reported	A (2 h) B (1:30) C (25 °C)	24.80	[22]
Ultrasonic-assisted extraction	Extraction temperature (A) Extraction time (B) Solid–liquid ratio (C)	Single factor test and Response surface method	95% ethanol Extraction times (2); Centrifugation (8000 rpm, 4 min)	A > B > C	A (65 °C) B (22 min) C (1:22)	12.13	[48]
	Solid–liquid ratio (A) Ultrasonic power (B) Extraction time (C)	Single factor test and Response surface method	The solvent is water	B, C > A	A (1:19) B (460 W) C (23 min)	60.15	[49]
	Extraction temperature (A) Extraction time (B) Solid–liquid ratio (C)	Not Reported	Ultrasonic power (198 W)	Not Reported	A (74 °C) B (27 min) C (1:103)	GLP80-1 4.34	[34]
Enzymatic extraction	Cellulase (A) Enzymatic hydrolysis time (B) Solid–liquid ratio (C)	Response surface method	Enzymatic hydrolysis temperature (70 °C); Ultrasonic power (210 W)	A > C > B	A (3%) B (3 h) C (1:30)	22.04	[40]
Microwave-assisted Extraction	Solid–liquid ratio (A) Microwave processing (B) Microwave power (C)	Univariate investigation Orthogonal design method	Extraction times (3) Soak (30 min)	Not Reported	A (1:30) B (100 S) C (800 W)	39.30	[36,50]
Multi-method joint extraction	Cellulase (A) Enzymatic hydrolysis time (B) Extraction time (C) Extraction temperature (D) Solid–liquid ratio (E)	Univariate investigation Orthogonal design method	Ultrasonic power (210 W)	Not Reported	A (2%) B (1.87 h) C (40.8 min) D (65 °C) E (1:30)	40.60	[26,41]

**Table 2:** Relative molecular mass, glycosidic bond type and monosaccharide composition of GLPs.

Name	Naming Rules	Method	Column Chromatography	Relative Molecular Mass	Monosaccharide Composition	Configuration Glycosidic Bonds	References
GRP-1	GRP is eluted with water to obtain GRP-1	Water extraction and alcohol precipitation	DEAE—52, Sephadex G—75	2.301 × 10^4^	Man, GlcA, Rha, Glc, Gal, Ara = 81.86:0.12:0.17:1259.7:0.54:0.33	Contains an α-glycosidic bond-linked	[63]
GRP-2	GRP is eluted with 0.05 mol/L NaCl to obtain GRP-2	Water extraction and alcohol precipitation	DEAE—52, Sephadex G—75	Not Reported	Man, GlcA, Rha, Glc, Gal, Fuc = 24.15:0.4:0.46:541.3:8.25:2.50	Not Reported	[63]
GRP-3	GRP is eluted with 0.1 mol/L NaCl to obtain GRP-3	Water extraction and alcohol precipitation	DEAE—52, Sephadex G—75	Not Reported	Man, GlcA, Rha, Glc, Gal, Ara, Fuc = 94.7:1.23:0.48:110:2.73:0.8:13.4	Not Reported	[63]
GLP80-1	After purification, GLP was named as GLP-80	ultrasonic-assisted extraction and ethanol precipitation	(DEAE)-cellulose 52; Sephadex G—100	1.63 × 10^4^	Glc, GlcA, Gal, Ara = 0.91:0.04:0.03:0.02	(1 → 4)-α-D-Glc, (1 →4,6)-α-D-Glc, (1→)-α-D-Glc	[34]
GLP	Named after the polysaccharide of GL	Water extraction and alcohol precipitation	DEAE-cellulose 52, Sephadex G-100	1.37 × 10^5^	Glc	the main chain linkage was → 4)-α-D-Glcp-(1→glycosidic bond, the terminal group α-D-Glcp-(1→ through →4,6)-α-D-Glcp-(1→ O-6 bond was connected to the main chain	[55]
GLP90-2	GL extracted with ethanol as GL-90. GL90 is eluted with 0.1 mol/L NaCl to obtain GLP90-2	Water extraction and alcohol precipitation	DEAE-FF, Sephadex G-75	7.76 × 10^3^	Ara	α-l-Araf-(1→,→5)-α-l-Araf-(1→,→3,5)-α-l-Araf-(1→,→3,5)-α-l-Araf-(1→)	[42]
GRP	Named after the polysaccharide of GL	Water extraction and alcohol precipitation	DEAE—52, Sephadex G—75	1.33 × 10^4^	Glc	α-D-glucan, (1→6)-linked and (1 →3)-linked backbone with a branch of one (1→6)linked and one terminal glucoses submitting at the C-4 position every fourteen residues	[2]
GL-100	GLP is eluted with water to obtain GL-100	Water extraction and alcohol precipitation	DEAE—32, Sephadex G—200	7.90 × 10^4^	Glc	α-(1→4) glycosidic bond-linked α-(1→4) glycosidic bond-linked glucan	[70]
GL-103	GLP is eluted with 0.05 mol/L phosphoric acid buffer solution to obtain GL-103	Water extraction and alcohol precipitation	DEAE-32, Sephadex G—200	7.00 × 10^4^	Glc	α-(1→4) glycosidic bond-linked α-(1→4) glycosidic bond-linked glucan	[70]
GL-120	GLP is eluted with 0.15 mol/L phosphoric acid buffer solution to obtain GL-120	Water extraction and alcohol precipitation	DEAE-32	Not Reported	Not Reported	Not Reported	[70]
GL-122	GLP is eluted with 0.2 mol/L NaCl to obtain GL-122	Water extraction and alcohol precipitation	DEAE-32	Not Reported	Not Reported	Not Reported	[70]
GLP-E	Enzyme extraction process of GL	enzymatic extraction	DEAE-52	4.66 × 10^6^, 3.77 × 10^5^, 1.04 × 10^4^	GlcA, Glc, Gal, Ara	Not Reported	[40]
GLP-E1	GLP-E is eluted with water to obtain GLP-E1	enzymatic extraction	DEAE-52	4.22 × 10^6^, 3.78 × 10^5^	GlcA, Glc, Ara	Not Reported	[40]
GLP-E2	GLP-E is eluted with 1 mol/L NaCl to obtain GLP-E2	enzymatic extraction	DEAE-52	4.63 × 10^6^, 1.02 × 10^4^	Glc	Not Reported	[40]
GLP-D1	GLP is eluted with water to obtain GLP-D1	Water extraction and alcohol precipitation	DEAE-52	Not Reported	Glc	Not Reported	[71]
GLP-D2	GLP is eluted with 0.05 mol/L NaCl to obtain GLP-D2	Water extraction and alcohol precipitation	DEAE-52	Not Reported	Rha, Ara, Xyl, Glc, Gal = 16.58:5.4:1:104.24:7.75	Not Reported	[71]
GLP-30	Named according to different concentrations of ethanol extracts. The final concentration of ethanol is 30%	Water extraction and alcohol precipitation	Not Reported	6.75 × 10^5^, 1.22 × 10^5^, 5.08 × 10^5^	Glc, Gal, Ara, GalA	Not Reported	[72]
GLP-50	Named according to different concentrations of ethanol extracts. The final concentration of ethanol is 50%	Water extraction and alcohol precipitation	Not Reported	4.95 × 10^5^, 1.37 × 10^5^	Glc, Gal, Ara, GalA	Not Reported	[72]
GLP-70	Named according to different concentrations of ethanol extracts. The final concentration of ethanol is 70%	Water extraction and alcohol precipitation	Not Reported	4.26 × 10^5^	Man, GalA, Glc, Gal, Ara,	Not Reported	[72]

Note: The “→” in the structural representations denote the glycosidic linkage patterns between monosaccharide residues.

**Table 3:** Primary structure of GLPs.

Method	Instruments	Tructural Information	Ref.
Complete acid hydrolysis	GC, HPLC	Monosaccharide composition and ratio	[67,71]
periodate oxidation	UV-Vis	Types and ratios of glycosidic bonds	[67]
Smith Degradation	GC GC-MS	Determination of the type of glycosidic bond	[67,73]
partial acid hydrolysis	HPAEC, MALDI-TOF-MS	Composition of main and branch chains	[67]
Methylation assay	GC-MS, NMR	Sugar ring, sugar residue linkage	[55,60]
Not Reported	FT-IR	Judgement of Characteristic Groups	[72]
Not Reported	NMR	Heterodimeric conformation, sugar residue linkage order	[60,67]
Not Reported	HPGPC, GPC	Molecular weight size and distribution	[40,63]

**Table 4:** The biological activities of GLPs.

Pharmacological	Name	Dose of GLPs	Animal Model	Mode of Action	References
Immunomodulatory effect	GLP, GLP80-1	0.5~250 μg/mL	splenic lymphocyte and RAW264.7 cells	promote proliferation of mouse spleen lymphocytes and RAW 264.7 cells	[34,55]
Immunomodulatory effect	GLP	2000 μg/mL	lymphocyte	Inhibition of PHA, COA and PWM induced lymphocyte proliferation	[68]
antitumor activity	GLP90-2	100~400 μg/mL	transgenic zebrafish experiments RAW264.7 cells	It works with TLR-4, PD-1 and VEGF to activate immunity and inhibit angiogenesis, thus inhibiting tumor growth and spread	[42]
antitumor activity	GLP	40, 160, 380 μg/mL	lung cancer cell line A549 cells	GLP reduces PCNA expression, leading to S and G2/M phase cell cycle arrest, inhibition of cell proliferation, migration, and induction of apoptosis	[15]
immunomodulatory activity, certain anti-inflammatory and anti-tumor activity,	GRP	15.6~500 μg/mL	splenic lymphocyte, RAW264.7 cells, A549 cells	Promotes proliferation of splenic lymphocytes and RAW264.7 cells and inhibits proliferation of A549 cells	[2]
immunomodulatory activity	GRP-1	7.8~31.25 μg/mL	splenic lymphocyte	Promotes proliferation of splenic lymphocytes	[63]
immunomodulatory activity	GRP-2	7.8~15.625 μg/mL	splenic lymphocyte	Promotes proliferation of splenic lymphocytes	[63]
immunomodulatory activity	GRP-3	7.8~62.5 μg/mL	splenic lymphocyte	Promotes proliferation of splenic lymphocytes	[63]

## Data Availability

All data included in this article are available from the corresponding author upon request.

## Abbreviations

**Abbreviation**	**Full name**
GL	Glehnia littoralis
GLPs	Polysaccharides from glehnia littoralis
CNKI	China national knowledge infrastructure
TCM	Traditional chinese medicine
NaOH	Sodium hydroxide
H_2_O_2_	Hydrogen peroxide
DEAE	Diethylaminoethyl
IC	Ion chromatography
FTIR	Fourier transform infrared spectroscopy
HPLC	High performance liquid chromatography
HPGPC	High performance gel permeation chromatography
GC	Gas chromatography
GC-MS	Gas chromatography-mass spectrometry
NMR	Nuclear magnetic resonance
UV	Ultraviolet
UV-Vis	Ultraviolet absorption spectroscopy
GPC	Gel permeation chromatography
HPSEC	High performance size exclusion chromatography
M*w*	Molecular weight
PDI	Polydispersity index
HPSEC-MALLS-RID	High performance size exclusion chromatography-multi-angle laser light scattering-refractive index detector
HPAEC	High performance anion exchange chromatography
MALDI-TOF-MS	Matrix-assisted laser desorption/ionization time-of-flight mass spectrometry
IR	Infrared spectroscopy
FT-IR	Fourier transform infrared spectrometer
CE	Capillary electrophoresis
TLC	Thin layer chromatography
RSM	Response surface methodology
SAR	Structure–activity relationship
DES	Deep eutectic solvent
MS	Mass spectrometry
H-HCOSY	Homonuclear correlation spectroscopy
HILICLC-MS	Hydrophilic interaction chromatography-liquid chromatography-mass spectrometry
Glc	Glucose
Man	Mannose
GlcA	Glucuronic acid, gluconic acid
Gal	Galactose
Ara	Arabinose
Rha	Rhamnose
Xyl	Xylose
Fuc	Fucose
RAW 264.7	Mouse mononuclear macrophage leukemia cells
NK cell	Natural killer cell
TNF-α	Tumor necrosis factor-α
NO	Nitric oxide
MTT	3-(4,5-dimethyl-2-thiazolyl)-2,5-diphenyl-2-H-tetrazolium bromide
A549 Cells	Human Non-Small Cell Lung Cancer Cells
PCNA	Proliferating cell nuclear antigen
PD-1	Programmed cell death protein-1
VEGF	Vascular endothelial growth factor
VEGFR-2	Vascular endothelial growth factor receptor 2
SPR	Surface plasmon resonance
IgG	Immunoglobulin G
IgM	Immunoglobulin M
IL-6	Interleukin-6
LPS	Lipopolysaccharide
ANN	Artificial Neural Networks
prep-UHPLC	Preparative ultra-high performance liquid chromatography
SFE	Supercritical fluid extraction

## References

[1] Jing Y.S., Zhang R.J., Wu L.F., Zhang D.S., Zheng Y.G. (2020). Structural characteristics and antioxidant activity of polysaccharide-iron complex from Glehniae Radix. Int. J. Food Prop..

[2] Du B.X., Fu Y.P., Wang X., Jiang H.Q., Lv Q.T., Du R.K., Yang Y., Rong R. (2019). Isolation, purification, structural analysis and biological activities of water-soluble polysaccharide from Glehniae radix. Int. J. Biol. Macromol..

[3] Lu C., Zhang S., Lei S.S., Wang D., Peng B., Shi R., Chong C.M., Zhong Z., Wang Y. (2024). A comprehensive review of the classical prescription Yiguan Jian: Phytochemistry, quality control, clinical applications, pharmacology, and safety profile. J. Ethnopharmacol..

[4] Zhang S., Cheng F., Yang L., Zeng J., Han F., Yu X., Zhu Y., Zhong G., He J. (2020). Chemical constituents from *Glehnia littoralis* and their chemotaxonomic significance. Nat. Prod. Res..

[5] Seo U.M., Zhao B.T., Kim Y.H., Kang J.S., Son J.K., Woo M.H. (2016). Simultaneous analysis of seven marker compounds from Saposhnikoviae Radix, Glehniae Radix and Peucedani Japonici Radix by HPLC/PDA. Arch. Pharm. Res..

[6] Dong Q., Yuan Y., Zhou Y., Zhang Y.X., Zhang J.P., Yu H.B., Jiao B.H., Liu X.Y., Lu X.L. (2018). Biotransformation of total coumarins of Radix Glehniae by Lecanicillium attenuatum W-1-9. J. Asian Nat. Prod. Res..

[7] Yang M., Li X., Zhang L., Wang C.C., Ji M.Y., Xu J.P., Zhang K.Y., Liu J.C., Zhang C.H., Li M.H. (2019). Ethnopharmacology, Phytochemistry, and Pharmacology of the Genus Glehnia: A Systematic Review. Evid.-Based Compl. Alt..

[8] Wang M., Zhu P., Zhao S., Nie C., Wang N., Du X., Zhou Y. (2017). Characterization, antioxidant activity and immunomodulatory activity of polysaccharides from the swollen culms of Zizania latifolia. Int. J. Biol. Macromol..

[9] Wang J., Zhang L., Yu Y., Cheung P.C. (2009). Enhancement of antitumor activities in sulfated and carboxymethylated polysaccharides of Ganoderma lucidum. J. Agric. Food Chem..

[10] Bian Z., Zhang R., Zhang X., Zhang J., Xu L., Zhu L., Ma Y., Liu Y. (2023). Extraction, structure and bioactivities of polysaccharides from Rehmannia glutinosa: A review. J. Ethnopharmacol..

[11] Huang Y., Nan L., Xiao C., Ji Q., Li K., Wei Q., Liu Y., Bao G. (2019). Optimum preparation method for self-assembled pegylation nano-adjuvant based on rehmannia glutinosa polysaccharide and its immunological effect on macrophages. Int. J. Nanomed..

[12] Leung P.H., Zhao S., Ho K.P., Wu J.Y. (2009). Chemical properties and antioxidant activity of exopolysaccharides from mycelial culture of Cordyceps sinensis fungus Cs-HK1. Food Chem..

[13] Liu M., Wang C., Zhang H., Guo H., Kang L., Li H., Li K. (2024). A systematic review on polysaccharides from *Morinda officinalis* How: Advances in the preparation, structural characterization and pharmacological activities. J. Ethnopharmacol..

[14] Mukherjee S., Jana S., Khawas S., Kicuntod J., Marschall M., Ray B., Ray S. (2022). Synthesis, molecular features and biological activities of modified plant polysaccharides. Carbohydr. Polym..

[15] Wu J., Gao W., Song Z., Xiong Q., Xu Y., Han Y., Yuan J., Zhang R., Cheng Y., Fang J. (2018). Anticancer activity of polysaccharide from *Glehnia littoralis* on human lung cancer cell line A549. Int. J. Biol. Macromol..

[16] Liu Y.M., Liu B., Wang J.F., Feng Y.T., Miao N.F. (2005). The extraction on polysaccharide of Radix Glehniae and immuno-regulating effects on Yin deficiency mice. Chin. J. Biochem. Pharm..

[17] Gao J., Lin L., Sun B., Zhao M. (2017). A comparison study on polysaccharides extracted from Laminaria japonica using different methods: Structural characterization and bile acid-binding capacity. Food Funct..

[18] Chen H., Zeng J., Wang B., Cheng Z., Xu J., Gao W., Chen K. (2021). Structural characterization and antioxidant activities of Bletilla striata polysaccharide extracted by different methods. Carbohydr. Polym..

[19] Ji X.L., Yin M.S., Nie H., Liu Y.Q. (2020). A review of isolation, chemical properties, and bioactivities of polysaccharides from Bletilla striata. BioMed Res. Int..

[20] Chen F., Huang G.L. (2018). Extraction and antioxidant activities of cushaw polysaccharide. Int. J. Biol. Macromol..

[21] Pawlaczyk G.I., Balicki S., Wilk K.A. (2019). Effect of various extraction methods on the structure of polyphenolic-polysaccharide conjugates from *Fragaria vesca* L. leaf. Int. J. Biol. Macromol..

[22] Jing Y.S., Zhang D.S., Zhang R.J., Su L., Pan M.S., Sun S.S., Wu L.F., Zheng Y.G. (2017). Effect of different extraction methods on the properties and biological activity of polysaccharides from Radix Glehniae. Food Mach..

[23] Fang X., Gu S., Jin Z., Hao M., Yin Z., Wang J. (2018). Optimization of ultrasonic-assisted simultaneous extraction of three active compounds from the fruits of forsythia suspensa and comparison with conventional extraction methods. Molecules.

[24] Si J., Yang C., Chen Y., Xie J., Tian S., Cheng Y., Hu X., Yu Q. (2023). Structural properties and adsorption capacities of *Mesona chinensis* Benth residues dietary fiber prepared by cellulase treatment assisted by Aspergillus niger or Trichoderma reesei. Food Chem..

[25] Zhao J.L., Zhang M., Zhou H.L. (2019). Microwave-Assisted Extraction, Purification, Partial Characterization, and Bioactivity of Polysaccharides from Panax ginseng. Molecules.

[26] Zhang R.J., Jing Y.S., Zhang D.S. (2019). Optimization of Glehniae Radix polysaccharide extraction process, antioxidant activity and protective effect on H_2_O_2_ induced oxidative damage in PC12 cells. Chin. J. Pharmacol. Toxicol..

[27] Shen Y.X., Liu H.X., Li H.S., Ren B.R. (2016). Optimisation of aqueous extraction of crude polysaccharides from Glehniae Radix. Chin. Food Safe Mag..

[28] Wang B.l., Xu Y., Chen L.J., Zhao G.M., Mi Z.Y., Lv D.H., Niu J.F. (2020). Optimizing the extraction of polysaccharides from *Bletilla ochracea* Schltr. using response surface methodology (RSM) and evaluating their antioxidant activity. Processes.

[29] Jayapala N., Toragall V., Kumar G., Chaudhari S.R., Baskaran V. (2022). Preparation, characterization, radical scavenging property and antidiabetic potential of laminarioligosaccharides derived from laminarin. Algal Res..

[30] Li Y.C., Zhao M., Gomez L.P., Senthamaraikannan R., Padamati R.B., O’Donnell C.P., Tiwari B.K. (2021). Investigation of enzyme-assisted methods combined with ultrasonication under a controlled alkali pretreatment for agar extraction from *Gelidium sesquipedale*. Food Hydrocoll..

[31] Wang N., Shi N., Fei H., Liu Y., Zhang Y., Li Z., Ruan C., Zhang D. (2022). Physicochemical, structural, and digestive properties of pea starch obtained via ultrasonic-assisted alkali extraction. Ultrason. Sonochem.

[32] Wei E.W.W., Yang R., Zhao H.P., Wang P.H., Zhao S.Q., Zhai W.C., Zhang Y., Zhou H.L. (2019). Microwave-assisted extraction releases the antioxidant polysaccharides from seabuckthorn (*Hippophae rhamnoides* L.) berries. Int. J. Biol. Macromol..

[33] Feng Z.J., Zhang X.H., Zhang J.P., Shang X.H., Gao Y., Lu X.L., Liu X.Y., Jiao B.H. (2014). A new aromatic glycoside from *Glehnia littoralis*. Nat. Prod. Res..

[34] Jing Y.S., Zhang R.J., Ma Y.F., Zhang Y.W., Zheng Y.G., Wu L.F., Zhang D.S. (2021). Structural elucidation, anti-radical and immunomodulatory activities of polysaccharides from the roots of *Glehnia littoralis*. Nat. Prod. Res..

[35] Gao F.Y., Zhou C.C., Wang Z.H., Zhu W.W., Wang X., Liu G.J. (2023). Solid-oil separation of coal tar residue to reduce polycyclic aromatic hydrocarbons via microwave-assisted extraction. J. Environ. Manag..

[36] Zhou H.Y., Lv S. (2016). Microwave-assisted extraction and antioxidant activities of polysaccharides from radix glehniae. Food Res. Dev..

[37] Pan J., Shi Y., Zou J., Zhang X., Xin B., Zhai B., Guo D., Sun J., Luan F. (2024). Preparation technologies, structural features, and biological activities of polysaccharides from *Mesona chinensis* Benth.: A review. J. Ethnopharmacol..

[38] Park J.J., Lee W.Y. (2021). Anti-glycation effect of Ecklonia cava polysaccharides extracted by combined ultrasound and enzyme-assisted extraction. Int. J. Biol. Macromol..

[39] Pan S., Wu S. (2014). Cellulase-assisted extraction and antioxidant activity of the polysaccharides from garlic. Carbohydr. Polym..

[40] Jing Y.S., Zhang D.S., Zhang R.J., Han Y., Liu D.B., Zheng Y.G., Wu L.F. (2019). Study on the compound enzyme extraction process of Glehniae Radix and its physicochemical properties. Food Mach..

[41] Jing Y.S., Yuan X.R., Dai L.X., Zhang R.J., Zhang H., Zhang Y.G., Wu L.F. (2022). Optimization of cellulase synergistic ultrasonic-assisted extraction of polysaccharide from glehniae radix and its physicochemical properties and immunomodulatory activity. Sci. Technol. Food Ind..

[42] Liu W., Li K., Zhang H., Li Y., Lin Z., Xu J., Guo Y. (2024). An antitumor arabinan from *Glehnia littoralis* activates immunity and inhibits angiogenesis. Int. J. Biol. Macromol..

[43] Xiang M.R., Wang P.Z., Jiang H.Q., Gong L.L., Lv Q.T., Rong R. (2017). Optimization of hot water extraction method of *Glehnia littoralis* polysaccharides by central composite design and response surface methodology. Shandong J. Tradit. Chin. Med..

[44] Jin M., Zhao K., Huang Q., Xu C., Shang P. (2012). Isolation, structure and bioactivities of the polysaccharides from *Angelica sinensis* (Oliv.) Diels: A review. Carbohydr. Polym..

[45] Luan F., Ji Y., Peng L., Liu Q., Cao H., Yang Y., He X., Zeng N. (2021). Extraction, purification, structural characteristics and biological properties of the polysaccharides from *Codonopsis pilosula*: A review. Carbohydr. Polym..

[46] Panda B.C., Mondal S., Devi K.S., Maiti T.K., Khatua S., Acharya K., Islam S.S. (2015). Pectic polysaccharide from the green fruits of *Momordica charantia* (Karela): Structural characterization and study of immunoenhancing and antioxidant properties. Carbohydr. Res..

[47] Liu J., Guo Y., Sun J., Lei Y., Guo M., Wang L. (2024). Extraction methods, multiple biological activities, and related mechanisms of *Momordica charantia* polysaccharide: A review. Int. J. Biol. Macromol..

[48] Jing Y.S., Zhang D.S., Su L., Zhang R.J., Wu L.F., Zhang Y.G. (2018). Studies on the physicochemical properties and biological activities of *Glehnia littoralis* polysaccharides from different locations. Food Res. Dev..

[49] Piasecka I., Brzezińska R., Kalisz S., Wiktor A., Górska A. (2024). Recovery of antioxidants and oils from blackcurrant and redcurrant wastes by ultrasound-assisted extraction. Food Biosci..

[50] Huang H., Huang G. (2020). Extraction, separation, modification, structural characterization, and antioxidant activity of plant polysaccharides. Chem. Biol. Drug Des..

[51] Yang M.L., Ren W.J., Li G.Y., Yang P., Chen R., He H. (2022). The effect of structure and preparation method on the bioactivity of polysaccharides from plants and fungi. Food Funct..

[52] Qi H.Y., Zhang Z.P., Liu J.Q., Chen Z.Q., Huang Q.X., Li J., Chen J.J., Wang M.X., Zhao D.Q., Wang Z.Y. (2021). Comparisons of isolation methods, structural features, and bioactivities of the polysaccharides from three common Panax species: A review of recent progress. Molecules.

[53] Cohen G.H., Johnstone D.B. (1964). Extracellular polysaccharides of *Azotobacter vinelandii*. J. Bacteriol..

[54] Hu Z.Y., Zhou H.L., Li Y.P., Wu M.F., Yu M., Sun X.S. (2019). Optimized purification process of polysaccharides from *Carex meyeriana* Kunth by macroporous resin, its characterization and immunomodulatory activity. Int. J. Biol. Macromol..

[55] Jing Y.S., Li J.Y., Zhang Y.W., Zhang R.J., Zheng Y.G., Hu B.B., Wu L.F., Zhang D.S. (2021). Structural characterization and biological activities of a novel polysaccharide from *Glehnia littoralis* and its application in preparation of nano-silver. Int. J. Biol. Macromol..

[56] Qamar S., Torres Y.J.M., Parekh H.S., Robert Falconer J. (2021). Extraction of medicinal cannabinoids through supercritical carbon dioxide technologies: A review. J. Chromatogr. B Anal. Technol. Biomed. Life Sci..

[57] Liu B., Tan Z. (2022). Separation and Purification of *Astragalus membranaceus* Polysaccharides by Deep Eutectic Solvents-Based Aqueous Two-Phase System. Molecules.

[58] Ai X., Yu P., Li X., Lai X., Yang M., Liu F., Luan F., Meng X. (2023). Polysaccharides from *Spirulina platensis*: Extraction methods, structural features and bioactivities diversity. Int. J. Biol. Macromol..

[59] Zhang J., Zhao J., Liu G., Li Y., Liang L., Liu X., Xu X., Wen C. (2023). Advance in *Morchella* sp. polysaccharides: Isolation, structural characterization and structure-activity relationship: A review. Int. J. Biol. Macromol..

[60] Li K.X. (2021). Study on the Polysaccharides of *Cynanchum stauntonii* and *Glehnia littoralis*. Master’s Thesis.

[61] He C.B., Li L., Tang F.X., Xiong H.J. (2009). Isolation and structure characterization of polysaccharide from *Morinda officinalis* How. Chem. J. Chin. Univ..

[62] Zhang H., Li J., Xia J., Lin S. (2013). Antioxidant activity and physicochemical properties of an acidic polysaccharide from *Morinda officinalis*. Int. J. Biol. Macromol..

[63] Du B.X., Xiang M.R., Fu Y.P., Zhang J., Jiang H.Q., Rong R. (2018). Investigation of isolation, purification, structural identification and in vitro Immunological function of polysaccharides in Glehniae Radix. Chin. J. Exp. Tradit. Medl. Form..

[64] Jing Y.S., Zhang H., Cheng W.J., Zhang Y.M., Zhang D.S., Zheng Y.G. (2022). Research progress on extraction process, physicochemical properties and biological activity of polysaccharides from Glehniae Radix. J. Food Saf. Food Qual..

[65] Ren Y., Bai Y., Zhang Z., Cai W., Del Rio Flores A. (2019). The Preparation and Structure Analysis Methods of Natural Polysaccharides of Plants and Fungi: A Review of Recent Development. Molecules.

[66] Zhang R., Zhang X.X., Tang Y.X., Mao J.L. (2020). Composition, isolation, purification and biological activities of *Sargassum fusiforme* polysaccharides: A review. Carbohydr. Polym..

[67] Du B.X. (2019). Isolation, Purification, Structural Analysis and Biological Activities of Water-Soluble Polysaccharide from the Root of Glehnia littoralis. Master’s Thesis.

[68] Fang X.D., You M., Ying W.B., Sun Z.M., Shen P.Z., Shi Z.Y., Ye Q.W. (1986). The immunosuppressive activities of polysaccharides from *Glehnia littoralis* (bei sha shen). Acta Pharm. Sin..

[69] Luo S.Y., Jiang Y., Yu X.L., Liu J.L., Li B.N., Zhang K.Y., Wang S.L., XIE T. (2024). Research Progress on Structure Characteristics, Biological Activity, Structure-Activity Relationship and Product Development of Dendrobium Polysaccharides. Sci. Technol. Food Ind..

[70] Fang X.D., Sun Z.M., Ying W.B., You M., Shen P.Z., Shi Z.Y. (1987). Studies on the polysaccharide composition of Glehniae radix. Chin. Tradit. Pat. Med..

[71] Yu Q.H., Du B.X., Du Y.Q., Yang J., Ma Q.Y., Wen R., Rong R. (2020). Effects of isolation and purification of polysaccharides from *Beishenthus annuus* and their degradation by intestinal flora on the proliferation of immune cells in vitro. Chin. Tradit. Pat. Med..

[72] Jing Y.S., Jin S., Zhang D.S., Zhang R.J., Zhang F.F., Zhang Y.G., Wu L.F. (2020). Ethanol fractional purification, physicochemical properties and antioxidant activity of polysaccharides from *Glehnia radix*. Food Mach..

[73] Zhang Y., Gu M., Wang K.P., Chen Z.X., Dai L.Q., Liu J.Y., Zeng F. (2010). Structure, chain conformation and antitumor activity of a novel polysaccharide from *Lentinus edodes*. Fitoterapia.

[74] Zhang M., Tian X., Wang Y., Wang D., Li W., Chen L., Pan W., Mehmood S., Chen Y. (2018). Immunomodulating activity of the polysaccharide TLH-3 from Tricholomalobayense in RAW264.7 macrophages. Int. J. Biol. Macromol..

[75] Halliwell B. (2022). Reactive oxygen species (ROS), oxygen radicals and antioxidants: Where are we now, where is the field going and where should we go?. Biochem. Biophys. Res. Commun..

[76] Ng T.B., Liu F., Wang H.X. (2004). The antioxidant effects of aqueous and organic extracts of Panax quinquefolium, Panax notoginseng, *Codonopsis pilosula*, Pseudostellaria heterophylla and *Glehnia littoralis*. J. Ethnopharmacol..

[77] Li J.G., Li Q.D. (2011). Free radical scavenging of adenophora polysaccharides. China Brew..

[78] Mohanty S.K., Mallappa K.S., Godavarthi A., Subbanarasiman B., Maniyam A. (2014). Evaluation of antioxidant, in vitro cytotoxicity of micropropagated and naturally grown plants of *Leptadenia reticulata* (Retz.) Wight & Arn.—An endangered medicinal plant. Asian Pac. J. Trop. Med..

[79] Wang X.L., Li N., Li Y., Zhao Y.N., Zhang L., Sun Y.J., Ohizumi Y., Xu J., Guo Y.Q. (2022). A novel polysaccharide from *Paeonia lactiflora* exerts anti-tumor activity via immunoregulation. Arab. J. Chem..

[80] Kong C.S., Um Y.R., Im Lee J., Kim Y.A., Yea S.S., Seo Y. (2010). Constituents isolated from *Glehnia littoralis* suppress proliferations of human cancer cells and MMP expression in HT1080 cells. Food Chem..

